# Comparative health assessment of urban and non-urban free-ranging mule deer (*Odocoileus hemionus*) in southeastern British Columbia, Canada

**DOI:** 10.7717/peerj.4968

**Published:** 2018-06-20

**Authors:** Amélie Mathieu, Mark Flint, Patrick M. Stent, Helen M. Schwantje, Thomas E. Wittum

**Affiliations:** 1 The Wilds, Cumberland, OH, USA; 2 Department of Veterinary Preventive Medicine, College of Veterinary Medicine, The Ohio State University, Columbus, OH, USA; 3 British Columbia Ministry of Forests, Lands, Natural Resource Operations and Rural Development, Cranbrook, British Columbia, Canada; 4 British Columbia Ministry of Forests, Lands, Natural Resource Operations and Rural Development, Nanaimo, British Columbia, Canada

**Keywords:** Mule deer, Wildlife translocation, *Odocoileus hemionus*, Wildlife management, Health risk assessment, Cervidae

## Abstract

**Background:**

The provincial wildlife management agency, British Columbia Ministry of Forests, Lands, Natural Resource Operations and Rural Development, performed a translocation to control the urban mule deer (*Odocoileus hemionus*; uMD) overpopulation and supplement the declining non-urban mule deer (nuMD) population in the Kootenay region, British Columbia, Canada. The objectives of this cross-sectional study were to evaluate the health of the urban and nuMD populations by comparing pathogen exposure, body condition scores (BCS) and pregnancy rates, to characterize the health risks associated with the translocation and to investigate the role of infectious diseases in the decline of the nuMD deer population.

**Methods:**

Two hundred free-ranging mule deer were captured in urban and non-urban environments in the Kootenay region from 2014 to 2017. BCS and morphometric examinations were performed for each deer. Blood samples collected from each deer were tested for exposure to selected pathogens and pregnancy status.

**Results:**

Body condition scores averaged 3.4 on a five-point scale, was greater in nuMD, and significantly differed between years. Antibodies were detected for adenovirus hemorrhagic disease virus (38.4% (uMD 43.7%, nuMD 33.3%)), bluetongue virus (0.6% (uMD 1.2%, nuMD 0%)), bovine respiratory syncytial virus (8.4% (uMD 4.6%, nuMD 12.1%)), bovine viral diarrhea virus (1.1% (uMD 0%, nuMD 2.2%)), bovine parainfluenza-3 virus (27.0% (uMD 27.6%, nuMD 26.4%)), *Neospora caninum* (22.1% (uMD 24.4%, nuMD 19.7%)) and *Toxoplasma gondii* (8.2% (uMD 12.3%, nuMD 3.9%)). No antibodies against epizootic hemorrhagic disease virus were detected. Pregnancy rates did not differ between the two deer populations (90.7% (uMD 90.6%, nuMD 90.9%)). Exposure to *N. caninum* was associated with a reduced probability of being pregnant. uMD were more likely to be exposed to *T. gondii* than nuMD.

**Discussion:**

Comparison of BCS, pregnancy rates and pathogen exposure of uMD and nuMD showed that the health of the two populations did not significantly differ, suggesting uMD translocations do not pose a severe risk of pathogen transmission between mule deer populations and that these selected pathogens do not factor in the decline of the nuMD population. However, inclusion of additional health indicators and creation of a robust predictive disease model are warranted to further characterize the health of mule deer and the health risks associated with uMD translocations. These results should be considered as part of a formal risk assessment for future uMD translocations in southeastern British Columbia.

## Introduction

Wildlife translocations carry inherent health and disease transmission risks, and are recognized as an important driver of wildlife disease emergence (Cunningham, 1996; Daszak, Cunningham & Hyatt, 2001). By altering host–pathogen interactions, they can negatively affect the health of the translocated individuals and that of the resident animal populations as well as have negative conservation, economic and ecological repercussions (Carbyn & Watson, 2001; Lankester, 2001; Woodford, 2000; Woodford & Rossiter, 1993). To ensure a translocation project is beneficial, it is crucial that the health risk be critically evaluated prior to performing a wildlife translocation, and that the translocation be planned in light of the health risk analysis findings (Cunningham, 1996).

Over the past decade, a dramatic increase in urban mule deer (uMD) population density was observed in communities in the Kootenay region of southeastern British Columbia, Canada. In parallel, the non-urban mule deer (nuMD) population declined from 2011 to 2014 (Mule Deer Working Group, 2016). While the uMD population growth is likely linked to the abundance of forage, the restricted hunting due to urban bylaws and the scarcity of predators, the factors driving the nuMD population decline remain unknown. It has been proposed that declining quality and quantity, variations in predator–prey dynamics, and winter weather conditions are involved (Mule Deer Working Group, 2016). To simultaneously reduce the overabundant urban population and supplement the declining non-urban population, local wildlife management agencies translocated 85 uMD in a trial over a two-year period. This translocation project has potential health implications for the translocated uMD, the resident nuMD, as well as other sympatric wildlife species and domestic livestock present at or near the release sites. Ideally, the risk of pathogen transmission should be investigated prior to performing a wildlife translocation and the translocation should be planned in light of the disease risk analysis findings (Toyofuku, 2002). However, the decision to conduct the health assessment concurrent with the translocation was dictated by the provincial wildlife agency.

Little is currently known of the driving factors of mule deer health in the Kootenay region. We designed a cross-sectional study to characterize mule deer health based on selected health indicators including pathogen exposure, nutritional status and pregnancy rates. Pathogens were chosen based on their capacity to negatively affect deer health (epizootic hemorrhagic disease virus (EHDV), bluetongue virus (BTV), adenovirus hemorrhagic disease virus (AHDV), *Neospora caninum*) (Basso et al., 2014; Ditchfield, Debbie & Karstad, 1964; Dubey et al., 1996; Dulac et al., 1989, 1992; Lapointe et al., 1999; ODFW, 2003; Pybus, Ravi & Pollock, 2014; Soldati et al., 2004; Sorden, Woods & Lehmkuhl, 2000; WDFW, 2017; Woods et al., 1994, 1996). Furthermore, given the potential of deer to be infected with bovine pathogens, we tested for exposure to viruses of the bovine respiratory disease complex (BRDC) (bovine respiratory syncytial virus (BRSV), bovine viral diarrhea virus (BVDV), bovine parainfluenza-3 virus (PI3)) to characterize the pathogen transmission risk posed by uMD translocation to resident livestock (McMartin, Snodgrass & Corrigall, 1977; Morton, Evermann & Dietrich, 1990; Tessaro, Carman & Deregt, 1999; Van Campen et al., 1997). We also included testing for exposure to *Toxoplasma gondii* given its potential public health repercussions (Dubey et al., 2004; Sacks et al., 1983). A better characterization of mule deer health will help qualify the health risk associated with future translocation projects as well as help define the role of infectious diseases in the decline of the nuMD deer population. We hypothesized uMD have a lower pathogen exposure rate, a higher body condition score (BCS) mean and a higher pregnancy rate than nuMD.

## Materials and Methods

### Study area and animals

**Figure 1:**
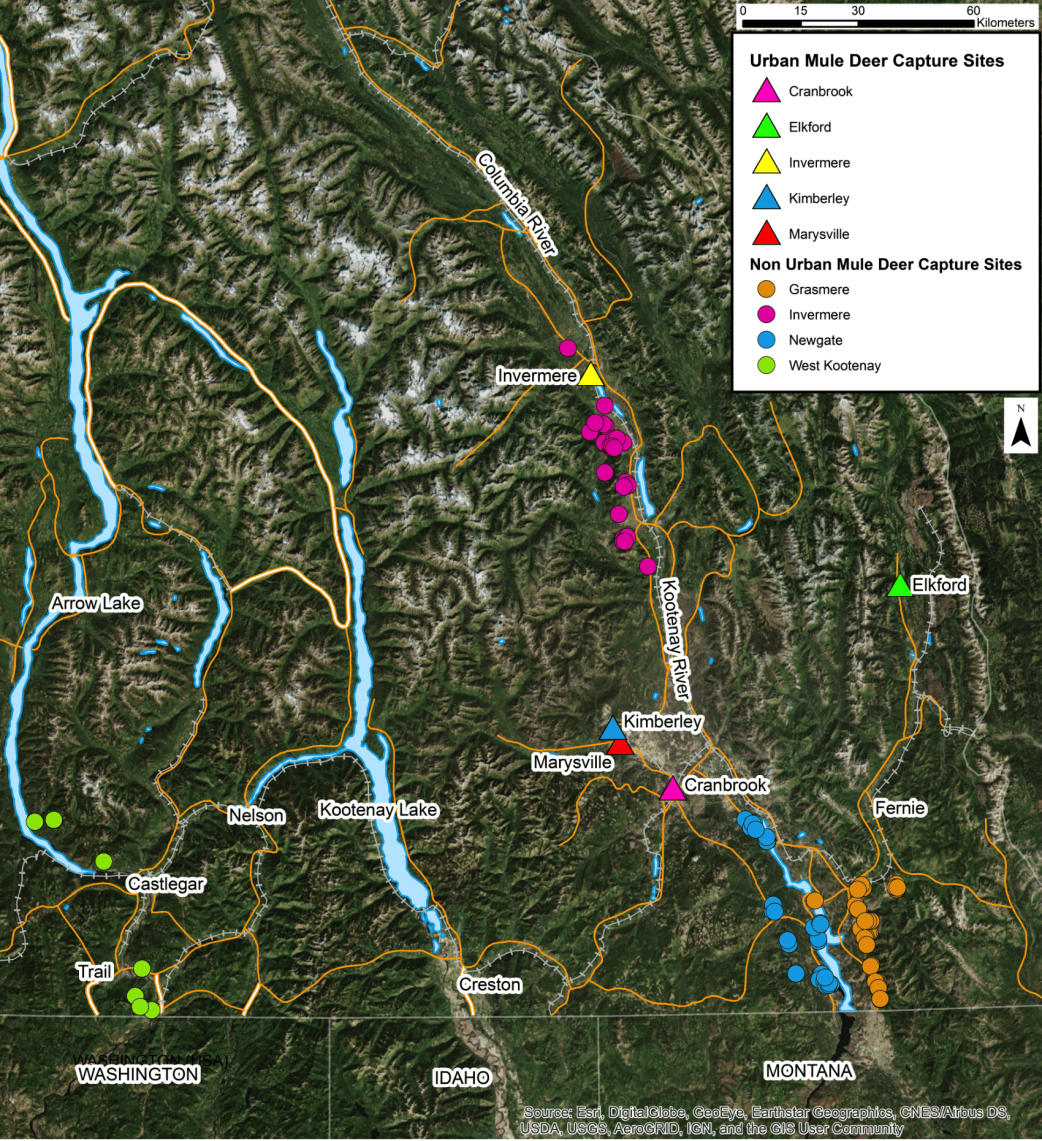
Location of capture sites of urban and non-urban of free-ranging mule deer (*Odocoileus hemionus*) in southeastern British Columbia, Canada.

This study was conducted in the Kootenay region of southeastern British Columbia, Canada. Deer were captured by the British Columbia Ministry of Forests, Lands, Natural Resource Operations and Rural Development (BC MFLNRORD) for two independent projects and sampled opportunistically for the present study (BC Wildlife Permit #CB16-224332 and #CB17-260952). Deer were captured in urban areas for translocation and in non-urban areas for radio collar fitting for ecological studies (Fig. 1). Deer were selected based on our ability to capture them safely and were only captured where permission had been granted. uMD were defined as deer captured inside of urban boundaries while nuMD were defined as deer captured outside of urban boundaries. Urban areas included Cranbrook (49°30′ to 49°32′N, 115°44′ to 115°46′W), Elkford (49°59′ to 50°1′N, 114°55′ to 114°55′W), Invermere (50°29′ to 50°30′N, 116°01′ to 116°03′W), Kimberley (49°40′ to 49°41′N, 115°58′ to 115°59′W) and Marysville (49°37′ to 49°38′N, 115°56′ to 115°57′W). These areas were typically characterized by residential areas, parks and green spaces. Non-urban areas included Grasmere (49°2′ to 50°2′N, 114°55′ to 116°26′W), Invermere (50°3′ to 50°33′N, 115°49′ to 116°8′W), Newgate (49°3′ to 49°27′N, 115°12′ to 115°31′W) and West Kootenay (49°0′ to 50°19′N, 115°58′ to 118°3′W). The non-urban areas were predominantly composed of montane forests and shrubland. GPS collar data collected from translocated uMD after completion of the present study revealed that post-translocation behavior varied greatly among individuals, and could be categorized as either migratory, non-migratory or wandering (Adams, 2018). No non-urban deer occupied urban habitats throughout the course of the study. Of the individuals that exhibited a migratory behavior following translocation, two returned to the community they were translocated from to overwinter the following year and migrated again to a non-urban summer range in spring. This suggests the urban deer population, at least in Invermere, contains some migratory animals that could come in contact with non-urban deer on summer and transitional ranges. The likelihood of animals comingling in the Newgate and Grasmere study areas was low considering the distance of nuMD and their seasonal ranges from communities (>20 km). Given the inability of determining whether a deer found in an urban area at the time of capture is a migratory nuMD deer overwintering in town or a non-migratory uMD, the definitions of uMD and nuMD were limited to where the individuals were captured, and the possibility of a small number of uMD and nuMD comingling was accepted as a limitation of this study.

Biometric data, including age, sex, BCS, was recorded post-capture. Age was estimated based on sequential development of dentition and wear, and categorized as follows: fawn, 0–12 months old; young adult, 13 months to three years old; adult, four to seven years old; aged adult, eight years and older (Severinghaus, 1949). Age classification data were excluded for 26 nuMD. Those animals were captured by independent capture teams and consistency in age estimates could not be confirmed. Sex was determined by observation of external genitalia. Body condition assessment was performed by a single observer (PMS) and was scored on a five-point scale (1—emaciated, 2—poor, 3—fair, 4—good, 5—excellent) (Shallow et al., 2015). Body condition scoring data was excluded for 15 uMD and 38 nuMD. Those animals were captured by independent capture teams and consistency in BCS assessment could not be confirmed. A subset of 28 deer (14 uMD, 14 nuMD) was weighed for the purpose of a coincident anesthesia study (Mathieu et al., 2017).

### Capture techniques

Captures happened sporadically between December 2014 and April 2017. For the purpose of this study, capture seasons were defined as: winter 2014–15, winter 2015–16, winter 2016–17. Non-urban MD were captured in December and January while uMD were captured in February and March. Animals were either darted and anesthetized from the ground, net gunned from a helicopter, or captured in Clover traps and anesthetized (Barrett, Nolan & Roy, 1982; Boesch et al., 2011; Clover, 1956). Deer were anesthetized with one of two drug protocols: medetomidine-azaperone-alfaxalone (Mathieu et al., 2017) or butorphanol-azaperone-medetomidine (Miller et al., 2009).

### Sample collection and testing

About 5–10 mL of blood was collected via jugular venipuncture and transferred to Vacutainer® plain blood collection tubes. Blood tubes were later centrifuged, and serum was extracted, aliquoted into cryovials and stored frozen (−20 °C) for two to three years.

For Pregnancy-Specific Protein B (PSPB) quantification using a sandwich ELISA that uses rabbit anti-moose PSPB polyclonal antibody coated plastic wells to bind cervid serum sample pregnancy-specific protein B (BioPRYN wild), serum samples were submitted to the BioTracking laboratory (BioTracking Inc., Moscow, ID, USA) (Duquette et al., 2012). Optical density (OD) cut-off value was set at OD > 0.21. For EHDV and BTV antibody titers determination using a competitive enzyme-linked immunosorbent assay (cELISA), serum samples were sent to the National Centre for Foreign Animal Disease (Canadian Food Inspection Agency, Winnipeg, MB, Canada) (CFIA, 2016a, 2016b). Samples with a percentage of inhibition of ≥40% were reported as positive. Positive samples were then serotyped using a serum neutralization test (CFIA, 2017). Sera were screened for evidence of cell toxicity at a final dilution of 1/10 after the addition of virus. For AHDV antibody titers determination using a deer adenovirus antigen specific ELISA, serum samples were submitted to the Oregon Veterinary Diagnostic Laboratory (College of Veterinary Medicine, Oregon State University, Corvallis, OR, USA). This assay detects serum IgG antibodies against a synthetic DAV spike antigen expressed in *Escherichia coli*. Methods and other assay details have not yet been published and are proprietary information of Drs Rob Bildfell and Ling Jin (College of Veterinary Medicine, Oregon State University). For BVDV, BRSV and PI3 antibody titers determination using virus neutralization tests, serum samples were submitted to the Abbotsford Animal Health Center Laboratory (Ministry of Agriculture Abbotsford Agricultural Centre, Abbotsford, BC, Canada) (OIE, 2017). Sera were screened for evidence of cell toxicity at a final dilution of 1/10 after the addition of virus. For *N. caninum* antibody titers determination using a cELISA, serum samples were sent to Prairie Diagnostic Services (Western College of Veterinary Medicine, University of Saskatchewan, Saskatoon, SK, Canada) (VMRD, 2017). Samples with a percent inhibition ≥30% were reported as positive. For *T. gondii* antibody titers determination using a commercial indirect ELISA optimized for use in cervids, serum samples were submitted to the Department of Veterinary Microbiology (Dr. Emily Jenkins) of the Western College of Veterinary Medicine (IDvet, 2013). Samples with a sample-to-positive percentage (S/P%) of ≥50% were reported as positive.

### Statistical analysis

Statistical analysis was performed using JMP 11 (SAS Institute Inc., Cary, NC, USA). Descriptive statistics were generated for the capture and biometric data. Normality of continuous outcome data was assessed using standard graphical methods (Dohoo, Martin & Stryhn, 2003). Weight, BCS, PSPB and pathogen prevalence means and their corresponding 95% Confidence Intervals were calculated for both uMD and nuMD populations.

A two-sample *t*-test was used to assess differences in mean weight between population types. The unadjusted associations between BCS and sex, population types and year were assessed using the non-parametric Mann–Whitney U test. BCS data for deer captured in winter 2014–15 were excluded from the temporal analysis of BCS since no uMD were sampled during that time.

PSPB results from samples collected prior to 40 days of gestation (i.e., prior to December 28th based on a mean conception date of November 18th (Dyer, Lincoln & Peatt, 1984)) were excluded from the statistical analysis. All animals for which an age class wasn’t specified were non-urban and sexually mature (young adult, adult or aged). Animals for which an age class was not defined were excluded for the age class analysis. All animals immobilized in winter 2014–15 were sampled prior to December 28th and were thus excluded from the PSPB analysis.

The effect of the independent variables, including age, population type, BCS, sampling year and exposure to pathogens, on the pregnancy status were evaluated using multivariable logistic regression models to control for potential confounding. Similarly, the effect of the independent variables, including age, population type, BCS, sampling year and pregnancy status and exposure to other pathogens, on each of the pathogen exposure outcomes were evaluated using multivariable logistic regression models. Logistic regression models were fitted for each pathogen exposure outcome independently using a forward selection procedure. Significant association were presented as adjusted odds ratios from the logistic regression models. Significance was set at *p* < 0.05.

## Results

### Animals sampled

A total of 200 mule deer were captured over three years. Data pertaining to age, sampling year, BCS, sex, capture technique and capture area distribution of the sampled animals is presented in Table 1.

**Table 1:** Characteristics of urban and non-urban free-ranging mule (*Odocoileus hemionus*) deer sampled in southeastern British Columbia, Canada.

	uMD *n* (%)	nuMD *n* (%)	Total *n* (%)
**Overall**	88 (44.0)	112 (56.0)	200 (100)
**Age**			
Fawn	20 (100)	0 (0)	20 (11.5)
Young adult	31 (47.0)	35 (53.0)	66 (37.9)
Adult	32 (42.1)	44 (57.9)	76 (43.7)
Aged adult	5 (41.7)	7 (58.2)	12 (6.9)
**Year**			
2014–15	0 (0)	42 (100)	42 (21.0)
2015–16	63 (57.3)	47 (42.7)	110 (55.0)
2016–17	25 (52.1)	23 (47.9)	48 (24.0)
**BCS**			
Emaciated	1 (100)	0 (0)	1 (0.7)
Poor	11 (64.7)	6 (35.3)	17 (11.6)
Fair	36 (61.0)	23 (39.0)	59 (40.1)
Good	20 (34.5)	38 (65.5)	58 (39.5)
Excellent	5 (41.7)	7 (58.3)	12 (8.2)
**Sex**			
Female	74 (39.8)	112 (60.2)	186 (93.0)
Male	14 (100)	0 (0)	14 (7.0)
**Capture technique**			
Darting	82 (77.4)	24 (22.6)	106 (53.0)
Net gunning	0 (0)	87 (100)	87 (43.5)
Clover trapping	6 (85.7)	1 (14.3)	7 (3.5)
**Urban areas**			
Cranbrook	25 (28.4)	NA	25 (28.4)
Elkford	15 (17.0)	NA	15 (17.0)
Invermere	14 (15.9)	NA	14 (15.9)
Kimberley	19 (21.6)	NA	19 (21.6)
Marysville	15 (17.0)	NA	15 (17.0)
**Non-urban areas**			
Invermere	NA	29 (25.9)	29 (25.9)
West Kootenay	NA	11 (9.8)	11 (9.8)
Grasmere	NA	39 (34.8)	39 (34.8)
Newgate	NA	33 (29.5)	33 (29.5)

### Biometrics

No physical abnormalities other than variations in body condition and dentition were noted on physical examination. Weights were documented for 28 female deer (14 uMD, 14 nuMD). Urban MD weighed 71.1 ± 6.6 kg (mean ± SD) and nuMD weighed 70.1 ± 9.6 kg. Young adults, adults and aged adults weighed 69.7 ± 9.3 kg, 72.8 ± 6.5 kg and 67.3 ± 3.9 kg, respectively. Mean weights did not differ significantly between populations (uMD: 71.1 ± 6.6 kg (95% CI [67.6–74.6] kg); nuMD: 70.2 ± 9.6 kg (95% CI [65.2–75.2] kg)) (*p* = 0.779). BCS did not significantly differ between females (3.4 ± 0.8 (95% CI [3.3–3.6]) and males (3.2 ± 0.8 (95% CI [2.8–3.6])) (*p* = 0.018), and data was thus pooled across sexes for all subsequent BCS analyses. BCS for both populations and all sampling years averaged 3.4 ± 0.8 (95% CI [3.3–3.6]), and significantly differed between populations (nuMD 3.6 ± 0.8 (95% CI [3.4–3.8]); uMD 3.2 ± 0.8 (95% CI [3.0–3.4]), *p* < 0.01) and sampling years (winter 2014–15: 3.8 ± 0.6 (95% CI [3.6–4.0]); winter 2015–16: 3.4 ± 0.8 (95% CI [3.2–3.6]); winter 2016–17: 3.0 ± 0.9 (95% CI [2.7–3.3]), *p* = 0.01). NuMD were found to be in overall better body condition than uMD. The difference was particularly evident in winter 2016–17 (uMD: 2.7 ± 0.6 (90% CI [2.4–2.9]; nuMD: 3.8 ± 1 (90% CI [3.1–4.2])) (*p* = 0.002). Moreover, uMD were in significantly poorer body condition in winter 2016–17 than in winter 2015–16 (3.5 ± 0.8 (90% CI [3.3–3.8]) (*p* < 0001). Mean BCS did not differ between uMD and nuMD sampled in winter 2015–16 (*p* = 0.08).

### Pregnancy-specific protein B quantification

Of the 108 females of reproductive age tested, 98 were deemed pregnant for an overall prevalence of 90.7% (95% CI [83.3–95.0]) (Table 2). Animals exposed to *N. caninum* were more likely to not be pregnant than animals not exposed to *N. caninum* (OR: 14.0, 95% CI [2.7–105.6]; *p* = 0.003). Pregnancy status was not significantly associated with the age class, sampling year, population type, BCS and exposure to all other pathogens tested.

**Table 2:** Prevalence of pregnancy based on Pregnancy-Specific Protein B levels in free-ranging mule deer (*Odocoileus hemionus*) in southeastern British Columbia, Canada.

		PSPB	
		*n*	% (95% CI)
**Overall**	–	108	90.7 (83.3–95.0)
**Age**	Young adult	32	84.4 (68.2–93.1)
	Adult/aged	53	96.2 (87.2–99.0)
**Year**	2015–16	67	88.1 (78.1–93.8)
	2016–17	30	96.7 (83.3–99.4)
**Population type**	Non-urban	33	90.9 (76.4–96.8)
	Urban	64	90.6 (81.0–95.6)
**BCS**	1–2	14	100 (78.5–100)
	3	32	90.6 (75.8–96.8)
	4	22	81.8 (61.5–92.7)
	5	7	100 (64.6–100)
**EHDV**	Positive	0	0 (NA)
	Negative	95	90.5 (83.0–94.9)
**AHDV**	Positive	48	93.8 (83.2–97.8)
	Negative	49	87.8 (75.8–94.3)
**BTV**	Positive	1	100 (20.6–100)
	Negative	94	90.4 (82.8–94.9)
**BRSV**	Positive	11	90.9 (62.3–98.4)
	Negative	97	90.7 (83.3–95.0)
**BVDV**	Positive	1	100 (20.6–100)
	Negative	107	90.6 (83.6–94.8)
**PI3**	Positive	35	85.7 (70.6–93.7)
	Negative	73	93.1 (84.9–97.0)
***N. caninum***	Positive	18	72.2 (49.1–87.5)
	Negative	75	97.3 (90.2–98.8)
***T. gondii***	Positive	11	72.7 (43.4–90.2)
	Negative	92	94.6 (87.9–97.6)

### Pathogen exposure

#### Epizootic hemorrhagic disease virus

About 176 deer were tested for exposure to EHDV. All results were negative (Table 3).

**Table 3:** Pathogen exposure prevalence in free-ranging mule deer (*Odocoileus hemionus*) in southeastern British Columbia, Canada.

		EHDV		AHDV		BTV		BRSV	
		*n*	% (95% CI)	*n*	% (95% CI)	*n*	% (95% CI)	*n*	% (95% CI)
**Overall**	–	176	0 (NA)	177	38.4 (31.6–45.8)	176	0.6 (0.1–3.1)	178	8.4 (5.2–13.4)
**Age**	Fawn	20	0 (NA)	20	20 (8.0–41.6)	20	0 (NA)	20	5 (0.9–23.6)
	Young adult	55	0 (NA)	56	33.9 (22.9–47.0)	55	0 (NA)	56	7.1 (2.8–17.0)
	Adult	68	0 (NA)	68	51.5 (39.8–62.9)	68	1.5 (0.3–7.9)	68	8.8 (4.1–17.9)
	Aged	9	0 (NA)	9	66.7 (35.4–87.9)	9	0 (NA)	9	0 (NA)
**Year**	2014–15	23	0 (NA)	23	21.7 (9.7–41.9)	23	0 (NA)	23	8.7 (2.4–26.8)
	2015–16	105	0 (NA)	106	35.8 (27.4–45.3)	105	0.9 (0.2–5.2)	107	7.5 (3.8–14.1)
	2016–17	48	0 (NA)	48	52.1 (38.3–65.5)	48	0 (NA)	48	10.4 (4.5–22.2)
**Population type**	Non-urban	90	0 (NA)	90	33.3 (24.4–43.6)	90	0 (NA)	91	12.1 (6.9–20.4)
	Urban	86	0 (NA)	87	43.7 (33.7–54.1)	86	1.2 (0.2–6.3)	87	4.6 (1.8–11.2)
**BCS**	1–2	16	0 (NA)	17	52.9 (31.0–73.8)	16	0 (NA)	17	11.8 (3.3–34.3)
	3	53	0 (NA)	54	40.7 (28.7–54.0)	53	1.9 (0.3–9.9)	54	3.7 (1.0–12.5)
	4	46	0 (NA)	45	40 (27.0–54.5)	46	0 (NA)	45	8.9 (3.5–20.7)
	5	12	0 (NA)	12	33.3 (13.8–60.9)	12	0 (NA)	12	8.3 (1.5–35.4)
**EHDV**	Positive	–	–	0	–	0	–	0	–
	Negative	–	–	175	38.3 (31.4–45.7)	176	0.6 (0.1–3.1)	175	7.4 (4.4–12.3)
**AHDV**	Positive	67	0 (NA)	–	–	67	1.5 (0.3–8.0)	68	8.8 (41–17.9)
	Negative	108	0 (NA)	–	–	108	0 (NA)	109	8.2 (4.4–15.0)
**BTV**	Positive	1	0 (NA)	1	100 (20.6–100)	–	–	1	0 (NA)
	Negative	175	0 (NA)	174	37.9 (31.0–45.3)	–	–	174	7.4 (4.4–12.4)
**BRSV**	Positive	13	0 (NA)	15	40 (19.8–64.2)	13	0 (NA)	–	–
	Negative	162	0 (NA)	162	38.3 (31.1–45.9)	162	0.6 (0.1–3.4)	–	–
**BVDV**	Positive	2	0 (NA)	2	100 (34.2–100)	2	0 (NA)	2	0 (NA)
	Negative	173	0 (NA)	175	37.8 (30.9–45.1)	173	5.8 (0.1–3.2)	176	8.5 (5.2–13.6)
**PI3**	Positive	47	0 (NA)	48	54.2 (40.3–67.4)	47	2.1 (0.3–11.1)	48	2.1 (0.4–10.9)
	Negative	128	0 (NA)	129	32.6 (25.1–41.0)	128	0 (NA)	130	10.8 (6.5–17.3)
***N. caninum***	Positive	35	0 (NA)	35	28.6 (16.3–45.0)	35	0 (NA)	35	11.4 (4.5–26.0)
	Negative	121	0 (NA)	123	43.9 (35.4–52.7)	121	0.8 (0.1–4.5)	123	6.5 (3.3–12.3)
***T. gondii***	Positive	12	0 (NA)	13	53.8 (29.1–76.8)	12	0 (NA)	13	7.7 (1.4–33.3)
	Negative	145	0 (NA)	145	38.6 (31.1–68.9)	145	0.7 (0.1–3.8)	145	6.9 (3.8–12.2)

		BVDV		PI3		*N. caninum*		*T. gondii*	
		*n*	% (95% CI)	*n*	% (95% CI)	*n*	% (95% CI)	*n*	% (95% CI)
**Overall**	–	178	1.1 (0.3–4.0)	178	27.0 (21.0–33.9)	158	22.1 (16.4–29.2)	158	8.2 (4.9–13.6)
**Age**	Fawn	20	0 (NA)	20	0 (NA)	19	31.6 (15.4–54.0)	19	0 (NA)
	Young adult	56	0 (NA)	56	12.5 (6.2–23.6)	50	24 (14.3–37.4)	50	10 (4.3–21.4)
	Adult	68	1.5 (0.3–7.9)	68	39.7 (28.9–51.6)	63	17.5 (10.0–28.6)	61	8.2 (4.3–21.4)
	Aged	9	0 (NA)	9	77.8 (45.3–93.7)	8	37.5 (13.7–69.4)	8	37.5 (13.7–69.4)
**Year**	2014–15	23	0 (NA)	23	34.8 (18.8–55.1)	17	47 (26.2–69.0)	19	10.5 (2.9–31.4)
	2015–16	107	1.9 (0.5–6.6)	107	25.2 (18.0–34.2)	93	16.1 (10.0–24.9)	91	7.7 (3.8–15.0)
	2016–17	48	0 (NA)	48	27.1 (16.6–41.0)	48	25 (14.9–38.8)	48	8.3 (3.2–19.6)
**Population type**	Non-urban	91	2.2 (0.6–7.7)	91	26.4 (18.4–36.2)	76	19.7 (12.3–30.0)	77	3.9 (1.3–10.8)
	Urban	87	0 (NA)	87	27.6 (19.3–37.8)	82	24.4 (16.4–34.7)	81	12.3 (6.8–21.3)
**BCS**	1–2	17	5.9 (1.0–27.0)	17	47.1 (26.2–69.0)	17	35.3 (17.3–58.7)	16	0 (NA)
	3	54	0 (NA)	54	27.8 (17.6–40.9)	51	13.7 (6.8–25.7)	50	12 (5.6–23.8)
	4	45	0 (NA)	45	28.9 (17.7–43.4)	42	31 (19.1–46.0)	42	11.9 (5.2–25.0)
	5	12	0 (NA)	12	25 (8.9–53.2)	8	12.5 (2.2–47.1)	8	0 (NA)
**EHDV**	Positive	0	–	0	–	0	–	0	–
	Negative	175	1.1 (0.3–4.1)	175	26.8 (20.8–3.9)	156	22.4 (16.6–29.6)	156	22.4 (16.6–29.6)
**AHDV**	Positive	68	2.9 (0.3–4.1)	68	38.2 (27.6–50.1)	64	15.6 (8.7–26.4)	63	11.1 (5.5–21.2)
	Negative	109	0 (NA)	109	20.2 (13.7–28.7)	94	25.6 (18.7–36.3)	95	6.3 (2.9–13.1)
**BTV**	Positive	1	0 (NA)	1	100 (20.6–100)	1	0 (NA)	1	0 (NA)
	Negative	174	1.1 (0.3–4.1)	174	26.4 (20.4–33.4)	155	22.6 (16.7–29.8)	156	7.7 (4.4–13.0)
**BRSV**	Positive	15	0 (NA)	15	6.7 (1.2–29.8)	12	33.3 (13.8–60.9)	11	9 (1.6–37.7)
	Negative	163	1.2 (0.3–4.4)	163	28.8 (22.4–36.2)	146	21.2 (15.4–28.6)	147	8.1 (4.7–13.7)
**BVDV**	Positive	–	–	2	100 (34.2–100)	2	0 (NA)	2	0 (NA)
	Negative	–	–	176	26.1 (20.2–33.1)	156	22.4 (16.6–29.6)	156	8.3 (4.9–13.7)
**PI3**	Positive	48	4.1 (1.2–14.0)	–	–	44	20.4 (11.2–34.5)	44	13.6 (6.4–26.7)
	Negative	130	0 (NA)	–	–	114	22.8 (16.1–31.3)	114	6.1 (3.0–12.1)
***N. caninum***	Positive	35	0 (NA)	35	25.7 (14.2–42.1)	–	–	35	14.3 (6.3–29.4)
	Negative	123	1.6 (0.4–5.7)	123	28.4 (21.2–37.0)	–	–	121	6.6 (3.4–12.5)
***T. gondii***	Positive	13	0 (NA)	12	46.2 (23.2–70.8)	13	38.5 (17.7–64.5)	–	–
	Negative	145	1.4 (0.4–4.9)	145	26.2 (19.7–33.9)	143	21 (15.1–28.4)	–	–

#### Bluetongue disease virus

Of the 176 deer tested for exposure to BTV, one was positive, for an overall prevalence of 0.6% (95% CI [0.1–3.1]) (Table 3). This sample had neutralizing antibodies to BTV-10 and BTV-17, and was negative for BTV-2, -8, -11 and -13.

#### Adenovirus hemorrhagic disease virus

Of the 177 deer tested for exposure to AHDV, 68 were positive for an overall prevalence of 38.4% (95% CI [31.6–45.8]) (Table 3). Exposure to AHDV was significantly associated with age, with older animals more likely to be exposed than younger animals (*p* = 0.014). Compared to fawns, young adults, adults and aged adults were 2.5 (95% CI [0.8–9.5]), 5.1 (95% CI [1.6–19.7]) and 8.9 (95% CI [1.6–63.0]) times more likely to be exposed to AHDV, respectively. Exposure to AHDV was significantly associated with the sampling year (*p* = 0.032), with animals sampled in winter 2016–17 more likely to be exposed than animals sampled in winter 2014–15 (OR: 3.9, 95% CI [1.3–13.4]; *p* = 0.013). Animals exposed to PI3 were more likely to be exposed to ADHV (OR: 2.4, 95% CI [1.2–4.8]; *p* = 0.009). Exposure to AHDV was not significantly associated with the population type, BCS and exposure to all other pathogens tested.

#### Bovine respiratory syncytial virus

Of the 178 deer tested for exposure to BRSV, 15 were positive for an overall prevalence of 8.4% (95% CI [5.2–13.4]) (Table 3). Exposure to BRSV was not significantly associated with the age, sampling year, population type, BCS and exposure to all other pathogens tested.

#### Bovine viral diarrhea virus

Of the 178 deer tested for exposure to BVDV, two were positive for an overall prevalence of 1.1% (95% CI [0.3–4.0]) (Table 3).

#### Bovine parainfluenza-3 virus

Of the 178 deer tested for exposure to PI3, 48 were positive for an overall prevalence of 27.0% (95% CI [21.0–33.9]) (Table 3). Exposure to PI3 was not significantly associated with the age class, sampling year, population type, BCS and exposure to all other pathogens tested.

#### Neospora caninum

Of the 158 deer tested for exposure to *N. caninum*, 35 were positive for an overall prevalence of 22.1% (95% CI [16.4–29.2]) (Table 3). Exposure to *N. caninum* was significantly associated with the sampling year, with animals sampled in winter 2014–15 more likely to be exposed than animals sampled in winter 2015–16 (OR: 4.6, 95% CI [1.5–14.1]; *p* = 0.12). Exposure to *N. caninum* was not significantly associated with the age class, population type and BCS.

#### Toxoplasma gondii

Of the 158 deer tested for exposure to *T. gondii*, 13 were positive for an overall prevalence of 8.2% (95% CI [4.9–13.6]) (Table 3). uMD were more likely to be exposed to *T. gondii* than nuMD (OR: 3.5, 95% CI [1.0–16.0]; *p* = 0.047).

## Discussion

Knowledge of the health status of a wild animal population and of its determinants and comparison of health data over time and with other wildlife populations can assist wildlife managers in planning conservation management actions such as translocations as well as to inform the use of other management tools. This study compared nutritional status, pregnancy rates and pathogen exposure of uMD and nuMD in the Kootenay region over three years. Based on the present findings, it appears the health of the two mule deer populations does not significantly differ, suggesting uMD translocations do not pose a severe risk of selected pathogen transmission between mule deer populations. Comingling of uMD and nuMD in some areas likely contributes to exchange of pathogens and may explain why both deer populations have similar exposure levels to most pathogens. Furthermore, the lack of significant difference in the measured health indicators of the two mule deer populations suggest that the pathogens screened for in this study do not play a major role in the decline of the nuMD population. The reasons for the recent decline of the nuMD population necessitate further investigation (Mule Deer Working Group, 2016).

### Biometrics

Overall, body condition varied from emaciated to excellent, with an average score of 3.4 on a five-point scale. Scores significantly differed between population types and sampling year. Non-urban MD were in overall better body condition than uMD. This difference in body condition is attributable to timing of captures and variations in winter conditions. Most nuMD were captured in early winter (December and January) whereas all uMD were captured in late winter (February and March) when fat stores are typically depleted. Winter severity may have also contributed to lower BCS score of uMD in 2016–2017 as southeastern British Columbia experienced the harshest winter in 20 years, with below average temperatures and above average snowfall from December to February (BC MFLNRO 2017).

Mule deer are generally a highly prolific species with reports of pregnancy rates ranging from 78.8% to 100% (Andelt, Pojar & Johnson, 2004; Chattin, 1948; Dyer, Lincoln & Peatt, 1984; Robinette & Gashwiler, 1950). The present study revealed an overall pregnancy rate of 90.7% and statistical analysis did not detect differences in pregnancy rates between populations or sampling years. Given this lack of difference in pregnancy rates between populations, it is unlikely that fertility is a viable explanation for differences in demographics between uMD and nuMD. Investigation of recruitment failure (e.g., poor fawn survival) as a possible explanation for the nuMD population decline is recommended.

### Pathogen exposure

Epizootic hemorrhagic disease (EHD) and bluetongue are seasonal arthropod-borne diseases caused by two orbiviruses. In Canada, sporadic EHD outbreaks in deer have been documented in southern portions of British Columbia, Alberta, Saskatchewan and Ontario (Ditchfield, Debbie & Karstad, 1964; Dulac et al., 1989, 1992; Pybus, Ravi & Pollock, 2014). EHD and BT outbreaks occur during late summer and early fall during hot and dry weather when animals congregate near water sources and come in contact with their vectors, the hematophagous midges of the genus *Culicoides* (Couvillion et al., 1981; Foster et al., 1977). Myers et al. (2015) and Dubay et al. (2004) observed an elevational difference in EHDV and BTV exposure in mule deer in Washington and Arizona with a greater antibody prevalence at lower elevations than at higher elevations. They attributed this difference to a greater presence of *Culicoides* sp. in dry shrub-steppe habitats with standing bodies of water compared to alpine, subalpine and forested areas. Also, *Culicoides* sp. are not known to overwinter in Canada, and the temporal pattern of outbreaks reflects the vector’s presence in the western provinces only during the months of August to October. It has been proposed that seasonal wind patterns move the infected midges northwards from endemic northern American areas into Southwestern Canada, or that the midges’ range is expanding northwards as a result of climate change (Purse et al., 2005; Sellers & Maarouf, 1991; Wittmann & Baylis, 2000). These observations are in agreement with the outbreaks in British Columbia that have been limited to the low elevation dry grasslands of the Okanagan Valley within 50 km of the American border. Given the viruses’ predilection for low elevation dry shrub-steppe habitats with standing bodies of water, it is unlikely the high elevation forested Kootenay region is a suitable habitat for EHDV and BTV, which is supported by the minimal exposure prevalence to either viruses in our population sample. We propose this remains true regardless of the season.

Adenovirus hemorrhagic disease (AHD), a highly fatal disease of mule deer and white-tailed deer, has been responsible for several deer mortality outbreaks in Iowa, California, Oregon and Washington (Lapointe et al., 1999; ODFW, 2003; Sorden, Woods & Lehmkuhl, 2000; WDFW, 2017; Woods et al., 1996). In Canada, AHDV was associated with the death of at least eight mule deer fawns in Waterton Lakes National Park in Alberta (T. Shury, 2018, personal communication). In the subsequent years, evidence of AHDV exposure was reported in elk (*Cervus canadensis*) in the East Kootenay region of British Columbia (H. Schwantje, 2018, personal communication) Older animals were more likely to be exposed than younger animals. This is in accordance with observations that AHD has a high mortality rate in fawns and that adults exposed to AHDV can recover (Boyce et al., 2000). The significance of the association between positive PI3 and AHDV seroprevalence is unknown and may have been the result of a statistical fluke. The high seroprevalence may be real or be a consequence of the unknown specificity of the assay. Further investigation is required to assess the specificity and sensitivity of the ELISA in mule deer, to establish the true prevalence of AHDV and to better understand its potential impact on the health of the translocated and resident animals.

Although evidence of exposure is commonly reported in wild cervids, the viruses of the BRDC are not considered significant pathogens of cervids (Dubay et al., 2015; Ingebrigtsen, Ludwig & McClurkin, 1986; Karstad, 1981; Myers et al., 2015; Roug et al., 2012; Thorsen & Henderson, 1971; Van Campen et al., 2001). Experimental infection with BVDV only resulted in mild diarrhea, coronitis and laminitis in reindeer, and did not clinically affect red deer, elk, mule deer or white-tailed deer (McMartin, Snodgrass & Corrigall, 1977; Morton, Evermann & Dietrich, 1990; Tessaro, Carman & Deregt, 1999; Van Campen et al., 1997). Further, ovine respiratory syncytial virus was experimentally shown to only induce asymptomatic pneumonic lesions in fawns (Bryson et al., 1988). However, we screened for these pathogens given the high likelihood of wildlife and livestock interactions in our study regions and the economic losses that could result from livestock contracting diseases from deer. No statistically significant differences in exposure to respiratory viruses between uMD and nuMD were detected. While this data is preliminary, it suggests that uMD translocation do not pose a significant risk of transmission of BRDC viruses to the resident livestock. However, screening of resident livestock for pathogen exposure and assessment of the true interactions between livestock and wildlife is warranted to better characterize the risk of pathogen transmission between mule deer and livestock.

The role of wildlife in the epidemiology of *N. caninum* and the impact of the pathogen on wildlife health are poorly understood, with most reports on *N. caninum* infection in wildlife consisting of seroprevalence studies in asymptomatic animals (Anderson et al., 2007; Dubey et al., 1999, 2009, 2008; Dubey & Thulliez, 2005; Ferroglio & Rossi, 2001; Lindsay, Little & Davidson, 2002; Malmsten, Jakubek & Bjorkman, 2011; Myers et al., 2015; Tavernier et al., 2015; Tiemann et al., 2005; Woods et al., 1994). In Canada, evidence of *N. caninum* exposure was reported in mountain and boreal caribou (*Rangifer tarandus caribou*) in British Columbia, in woodland caribou in the Northwest Territories and in elk in Alberta (Mathieu, Carlsson & Kutz, 2015; Pruvot et al., 2014; Schwantje et al., 2014, 2016; Sifton, 2005). The present study identified a relation between seropositivity to *N. caninum* and reduction in pregnancy rates, but no definitive link is suggested. Neosporosis in cervids has been associated with fawns born dead, weak or with severe neurologic deficits (Basso et al., 2014; Dubey et al., 1996; Soldati et al., 2004; Woods et al., 1994). With a prevalence of 22.1% and suspicion of detrimental reproductive effects, *N. caninum* likely is a determinant of mule deer health in the Kootenay region. However, given that exposure to *N. caninum* does not differ between uMD and nuMD, transmission of *N. caninum* through uMD translocations represents a low health risk for either deer population.

The protozoan parasite *T. gondii* can infect most warm-blooded animals and relies on a felid intermediate host to complete its life cycle. While serological evidence of *T. gondii* exposure is abundant, the clinical significance of *T. gondii* in free-ranging mule deer remains poorly characterized (Lindsay et al., 2005). The overall seroprevalence for *T. gondii* in our population sample was low, but uMD were shown to be more likely to be exposed to *T. gondii* than nuMD. This observation is consistent with other studies that investigated urbanization as a risk factor for *T. gondii* exposure in wildlife (Ballash et al., 2015; Conrad et al., 2005; Lehrer et al., 2010). The increased seroprevalence in deer across the urban gradient was proposed to result from the greater risk of exposure to oocysts shed by domestic cats given their higher population density within city limits (Ballash et al., 2015). Bobcats and cougars are likely contributors to the environmental contamination in the Kootenay region in both urban and non-urban environments (Aramini, Stephen & Dubey, 1998; Bevins et al., 2012). While of apparent limited concern for the health of mule deer, *T. gondii* poses a significant public health risk to individuals that may consume viable tissue cysts in raw or undercooked mule deer venison (Dubey et al., 2004; Sacks et al., 1983), and uMD translocation may result in an increased prevalence of *T. gondii* in non-urban areas.

### Study limitations

Interpretation of serology results must be done with caution. Circulating antibodies may result from an active or latent infection or from a past exposure to a pathogen. As such, detection of antibodies does not characterize the nature of the infection, but merely indicates exposure to a pathogen. Since only survivors are available for testing, diseases with a high fatality rate are likely under-represented in this study. Serological assays vary in sensitivity and specificity and most are only validated in domestic species. Of all the assays used in this study, only the AHDV, *T. gondii* and PSPB assays were validated in wildlife. PSPB may remain in circulation for several days to weeks after fetal death, resulting in false positives if sampling is done shortly following the loss of the fetus. In cattle, PSPB has a half-life of over seven days (Kiracofe et al., 1993). Given these limitations, interpretation of serologic results should be nuanced, with more importance given to trends than to exact values.

While assessment of body condition was exclusively done by PMS to maximize consistency of observations, this method is subject to some variability. As such, the data reported here only partially reflects the nutritional status of the Kootenay mule deer population.

Given limited funds and access to carcasses, this study did not investigate the effect of parasitism on mule deer health despite early evidence of *Echinococcus* sp. and lung worm infections in nuMD (Mathieu et al., 2017). The role of parasitism on mule deer health warrants further investigations.

The limited sample size obtained for some of the variables makes differences more difficult to detect statistically. However, strong associations, such as the ones reported for *N. caninum* exposure and reduction in pregnancy rates and for *T. gondii* exposure and urban area, should not be masked by a small sample size. For example, even though BCS did not vary significantly between males and females, this warrants further investigation as this difference may be due to the small number of males sampled and to the restricted age distribution of males.

## Conclusions

Body condition scores varied by sampling year and population type, pregnancy rates were overall high and antibodies were detected against AHDV, BTV, BRSV, BVDV, PI3, *N. caninum* and *T. gondii.* No antibodies against EHDV were detected. Exposure to AHDV, PI3 and *N. caninum* was common whereas exposure to BTV, BRSV, BVDV and *T. gondii* was rare. Exposure to *T. gondii* was more prevalent in the uMD. Given the many limitations of serology, inclusion of additional health indicators, such as blood biochemistry and hematology, trace mineral levels, parasite burden, and post-mortem examination findings of apparently healthy and diseased animals is advisable to better characterize mule deer health. The results of this study should be considered as part of a formal risk assessment for future uMD translocations in southeastern British Columbia.

## Supplemental Information

10.7717/peerj.4968/supp-1 Supplemental Information 1 Comparative health assessment of urban and non-urban free-ranging mule deer (*Odocoileus hemionus*) in the Kootenay region, British Columbia, Canada. “1” represents a positive result. “2” represents a negative result.

## Additional Information and Declarations

### Data Availability

The following information was supplied regarding data availability: The raw data are provided in a Supplemental File.
